# Characteristics of Trunk Acceleration and Angular Velocity in Turning Movement in Post-Stroke Patients with High Risk of Falling

**DOI:** 10.3390/s25092689

**Published:** 2025-04-24

**Authors:** Daiki Naito, Keita Honda, Yusuke Sekiguchi, Shin-Ichi Izumi, Satoru Ebihara

**Affiliations:** 1 Department of Rehabilitation Medicine, Tohoku University Graduate School of Medicine, 1-1 Seiryo-machi, Aoba-ku, Sendai 980-8574, Miyagi, Japan; keita.honda.0528@gmail.com (K.H.); yusuke.sekiguchi.b2@tohoku.ac.jp (Y.S.); shinichi.izumi.c2@tohoku.ac.jp (S.-I.I.); satoru.ebihara.c4@tohoku.ac.jp (S.E.); 2 Department of Rehabilitation, Southern Tohoku Second Hospital, 6-95 Yatsuyamada, Koriyama 963-8052, Fukushima, Japan; 3 Department of Rehabilitation, Kumamoto Health Science University, 325 Izumi-machi, Kita-ku, Kumamoto 861-5533, Kumamoto, Japan; 4 Graduate School of Biomedical Engineering, Tohoku University, 6-6-12 Aoba, Aramaki, Aoba-ku, Sendai 980-8579, Miyagi, Japan

**Keywords:** stroke, balance, TUG, IMU

## Abstract

**Highlights:**

**What are the main findings?**

- Post-stroke patients with a high fall risk showed significantly slower trunk rotation during turning movements compared to low-risk patients and healthy controls.
- Although movement speed was controlled during turning movement, high-risk patients exhibited increased trunk disturbance in the flexion direction.

**What is the implication of the main finding?**

- An assessment of trunk angular velocity during turning movements can provide valuable insights for target.
- Assessment of trunk kinematics during turning movement based on fall risk could contribute to enhancing postural control.

**Abstract:**

Although falls commonly occur in post-stroke patients during turning, the characteristics of trunk movement during turning in individuals at a high risk of falling remain unclear. The aim of this study was to clarify the characteristics of trunk translational and rotational movements during turning in post-stroke patients with a high risk of falling. Trunk acceleration and angular velocity were measured using the inertial measurement unit of an iPhone in the timed up and go test and compared among 13 post-stroke patients with a high risk of falling (age: 69.38 ± 12.44 years, Berg Balance Scale (BBS) < 45), 18 post-stroke patients with a low risk of falling (age: 71.22 ± 8.50 years, BBS ≥ 45), and 10 age-matched healthy controls (age: 65.90 ± 11.57). We examined the differences in trunk movement during turning between groups and the relationships between the BBS score and trunk movement. The high-risk group exhibited the longest completion time (χ^2^ = 31.21, *p* < 0.001) and the lowest maximum of trunk angular velocity along the vertical axis among groups (χ^2^ = 28.51, *p* < 0.001). Furthermore, the high-risk group showed a higher minimum (absolute value) of trunk angular velocity along the mediolateral axis compared to the low-risk group (χ^2^ = 9.80, *p* = 0.007). The maximum trunk angular velocity along the vertical axis (r = 0.66, *p* < 0.001) and the minimum trunk angular velocity along the mediolateral axis (r = 0.51, *p* = 0.003) were significantly correlated with the BBS score. We found that post-stroke patients with a high risk of falling exhibited slower trunk rotation angular velocity and faster trunk flexion angular velocity during turning compared to low-risk groups. Our findings suggest that despite the decrease during turning speed due to poor balance control, post-stroke patients with a high risk of falling exhibit a greater disturbance in the sagittal plane.

## 1. Introduction

Falls are a common problem among post-stroke patients, and fall injuries pose a risk for reduced quality of life in post-stroke patients [1]. Post-stroke patients experience falls more frequently than healthy older adults, particularly during walking [2,3,4]. Therefore, fall prevention is an important rehabilitation goal for post-stroke patients. Frequent falls in post-stroke patients are attributed to reduced body functions, including motor function, sensory function, and balance ability [4]. Motor and sensory paresis and impaired trunk function in post-stroke patients are related to balance disorders during walking [5,6,7].

Turning, as well as straight-line walking, is an important component of everyday mobility [8]. Compared to straight-line walking in healthy adults, turning requires a greater range of external rotation of the hip/internal rotation of the inner leg and increased foot pressure (i.e., weight bearing) on the inner leg [9]. Additionally, in healthy adults, the propulsive force generated by the outer leg leads to an increase in angular momentum in the transverse plane around the inner leg, which requires more complex control during turning compared to walking [10]. In fact, the rate of fall accidents was higher for turning than for straight walking [11]. Given the task complexity of turning and the high rate of falls during turning, it is necessary to clarify the characteristics of turning movements in post-stroke patients and to develop fall prevention strategies that address the specific challenges of turning movement disorder. However, many existing studies on post-stroke patients have focused on straight walking, with fewer reports on turning movements [12].

Differences during turning movements have been identified between post-stroke patients and healthy adults. Studies have reported that post-stroke patients take longer to complete turning movements compared to healthy adults [12,13]. Additionally, post-stroke patients exhibit reduced trunk angular velocity along the vertical axis and trunk acceleration in the antero-posterior direction compared to healthy adults, indicating a decrease in trunk movement speed during turning in post-stroke patients [13]. In other words, post-stroke patients might employ a more cautious strategy by slowing down their turning movements compared to healthy controls [14]. On the other hand, some studies have suggested that walking slowly is an inadequate strategy for reducing gait instability [15,16]. In previous studies focusing on straight-line walking, post-stroke patients with poor clinical balance scores showed greater lateral sway than those with good scores, despite their slower gait speeds [15,16]. Therefore, post-stroke patients with poor clinical balance scores may exhibit greater lateral sway even with a slower turning speed, potentially contributing to falls during turning movements. However, to our knowledge, it has been unclear in which direction post-stroke patients at a high risk for falls (i.e., poor balance ability) are unstable during turning movements.

**Figure 1:**
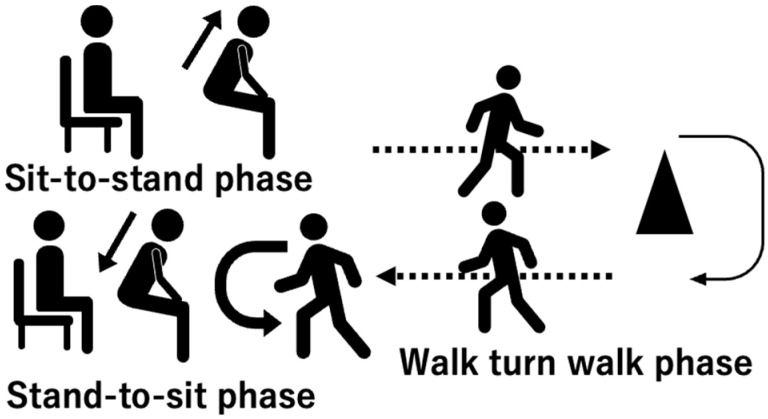
Sequence of the timed up and go test.

The aim of this study was to determine the differences in gait instability during turning movements between post-stroke patients at high and low risks of falling. Clarifying these relationships is expected to contribute to the development of rehabilitation programs designed to improve turning stability in post-stroke patients with poor balance ability, ultimately leading to safer performance of daily activities that frequently involve turning. In this study, we analyzed the characteristics of turning movements in the timed up and go test (TUG) (Figure 1). We hypothesized that (1) post-stroke patients with a high risk of falling exhibit smaller angular velocities along the vertical axis during turning movement than those with a low risk of falling, and (2) post-stroke patients with a high risk of falling exhibit greater disturbance in the mediolateral direction during turning movement than those with a low risk of falling.

## 2. Materials and Methods

### 2.1. Participants

A total of 31 post-stroke patients (19 males, age range of 40–89 years) admitted to our convalescent rehabilitation ward and 10 age-matched controls (6 males, age range of 40–80 years) were included in this study. The inclusion criteria for the post-stroke group were as follows: (1) hemorrhage or infarction in the supratentorial region on unilateral side by computed tomography or magnetic resonance imaging and diagnosis of cerebral hemorrhage or infraction, (2) patients who had received medical treatment after stroke onset, (3) patients aged 20 years old or older, and (4) patients who were able to perform TUG with cane and orthosis and without assistance. The inclusion criteria for age-matched controls also included (3) and (4). Exclusion criteria for post-stroke group and controls were (1) existing neurological (post-stroke group excludes cerebrovascular disorders treated in this trial), motor, circulatory, or respiratory diseases that interfere with the performance in the study, and (2) existing higher brain dysfunction, cognitive impairment, or aphasia that interferes with performance in the study. Participants provided written informed consent.

### 2.2. Clinical Assessment

Post-stroke patients used the Berg Balance Scale (BBS) as a balance ability assessment. Post-stroke patients with a BBS score below 45 were assigned to the high-risk group, and those with a BBS score of 45 or above were assigned to the low-risk group [17]. After allocation to the groups, post-stroke patients underwent Fugl-Meyer Assessment of Lower extremity (FMA-L) [18], used Stroke Impairment Assessment Set (SIAS) to determine scores for lower extremity motor function (hip flexion test, knee extension test, and foot tap test) [19], and used the Trunk Impairment Scale (TIS) [20].

### 2.3. Turning Movement Task

**Figure 2:**
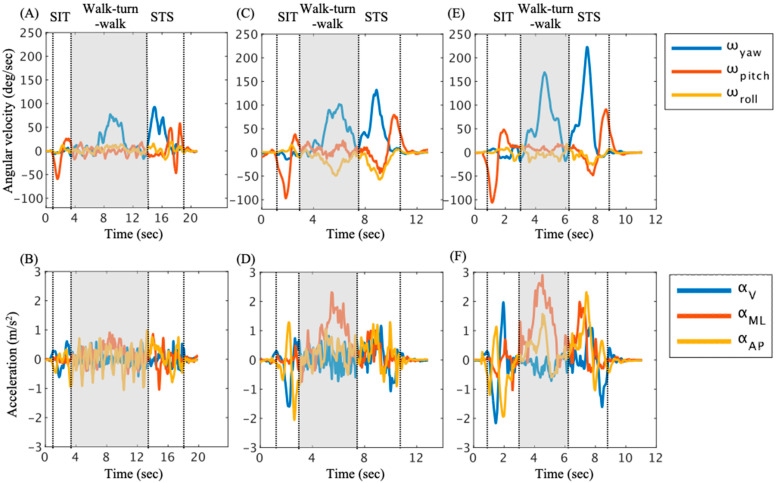
Representative trunk movement data during the timed up and go (TUG) test for the high-risk group of post-stroke patients (**A**,**B**), the low-risk group of post-stroke patients (**C**,**D**), and the age-matched controls (**E**,**F**). The gray area indicates the walk–turn–walk phase. The dotted vertical line on the left represents the start of the TUG test, and the dotted vertical line on the right represents the end of the TUG test. (**A**,**C**,**E**) The angular velocity of the trunk in all planes during the TUG test. (**B**,**D**,**F**) The acceleration of the trunk in all directions during the TUG test.

Turning movement was assessed using the instrumented TUG (iTUG: iOS application SENIOR Quality (Digital Standard Inc., Osaka, Japan)) [13] (Figure 1), which uses an IMU to measure trunk movement in the TUG (Figure 2). The TUG comprises three distinct phases: 1. Sit-to-stand phase: The participant transitions from a seated position in a standard chair to a standing position. This phase assesses the individual’s ability to rise from a seated posture, reflecting lower limb strength and postural control. 2. Stand-to-sit phase: Following a short walk, the participant returns to the chair and sits down. This phase evaluates the individual’s ability to safely and smoothly lower themselves into a seated position, indicating balance and coordination. 3. Walk–turn–walk phase: This central phase involves the participant walking a specified distance (typically 3 m), turning around a cone or marker, and walking back to the starting point. This segment assesses gait speed, dynamic balance, and turning ability, all of which are crucial for functional mobility.

The iOS application SENIOR Quality (Digital Standard Inc., Osaka, Japan) [21] and the iPhone 12 mini (Apple Inc., Cupertino, CA, USA) served as the inertial measurement unit (IMU). Previous studies using the same type of application demonstrated a relationship between the measured values and the severity of symptoms and mobility impairments in patients with normal pressure hydrocephalus. Therefore, the application used in this study has established validity for assessing mobility [21,22]. Furthermore, intraclass correlation coefficients were calculated for the results of this study, revealing high intra-rater reliability (Appendix A Table A1).

The iPhone 12 mini was placed in a pouch and positioned above the navel. SENIOR Quality recorded the following data: angular velocity along the vertical axis (ω_yaw_), mediolateral axis (ω_pitch_), and antero-posterior axis (ω_roll_) and acceleration in the antero-posterior direction (α_AP_), the vertical direction (α_V_), and the mediolateral direction (α_ML_). Positive values of ω_yaw_, ω_pitch_, and ω_roll_ indicated turning velocity toward the affected side, trunk extension velocity, and lateral flexion velocity toward the unaffected side in the group of post-stroke patients and turning velocity toward the left side, trunk extension velocity, and lateral flexion velocity to the right side in the control group. Positive values of α_V_, α_ML_, and α_AP_ indicated upward acceleration, lateral acceleration toward the unaffected side, and forward acceleration in the post-stroke patient group and upward acceleration, lateral acceleration toward the right side, and forward acceleration in the control group. The data were recorded at a sampling rate of 100 Hz.

In the iTUG, the subjects were instructed to perform the TUG at a comfortable speed. The subjects equipped with the IMU sat in the chairs (height 0.43 m) and prepared for the TUG start cue. At the start signal, subjects stood up and walked 3 m, turned around a cone, and returned to the chair and sat down. Post-stroke patients were instructed to turn toward the affected side, and the control group was instructed to turn toward the left side. Subjects performed the iTUG test three times and were allowed to use their usual cane and orthosis, as used in their daily lives.

### 2.4. Data Analysis

The data were smoothed, and noise was reduced using a 50 Hz moving average filter (MATLAB R2021b: MathWorks, Natick, MA, USA) [23]. This was carried out because the TUG test includes rapid movements, such as sit-to-stand transitions and turning, which can introduce high-frequency components into the data. The smoothed data were divided into the sit-to-stand phase (SIT), the walk–turn–walk phase, and the stand-to-sit phase (STS) [24] (Figure 2). The start of the SIT was determined by a ω_pitch_ value below −10 deg/s, and the end of the SIT was determined by a ω_pitch_ value below 10 deg/s [25]. The start of the STS was determined by a ω_yaw_ value above 10 deg/s, and the end of the STS was indicated by the max value of α_V_ immediately after the peak value of ω_pitch_ [26]. The walk–turn–walk phase was defined as the period from the start of the STS to end of the SIT [27].

The maximum, minimum, and root mean square (RMS) values of the acceleration and angular velocity were calculated for each phase. The RMS values indicated the variability of angular velocity and acceleration [28]. Data analysis was performed using MATLAB R2021b (MathWorks, Natick, MA, USA).

### 2.5. Statistical Analysis

Sex, diagnosis, paretic side, use of a cane and orthosis, and SIAS were compared between the high-risk group and the low-risk group using the Chi-square test. Time post-stroke, BBS, FMA-L, and TIS scores were compared between the high-risk and the low-risk groups using the Willcoxon rank sum test. Age, height, weight, trunk angular velocity and acceleration parameters, and the duration of the walk–turn–walk phase were compared among the high-risk group, low-risk group, and controls using the Kruskal–Wallis test, followed by Steel–Dwass multiple comparison procedures. The effect size and statistical power were calculated for each value. The effect size ($\eta^{2}$) was defined as follows: large (≥0.14), medium (≥0.06), and small (≥0.01) [29]. Sufficient statistical power (1 − β) was defined as ≥0.80 [29]. Using G power, the required sample size was calculated based on the obtained effect size, resulting in a sample size of 9 with a power (1 − β) of 0.88. Consequently, this study demonstrates both sufficient sample size and adequate statistical power.

Spearman’s correlation coefficient was used to analyze the association between the trunk IMU data, showing significant differences between the high-risk and low-risk groups and the SIAS lower extremity score. The correlation coefficients were classified as weak (0.10 ≤ r ≤ 0.39), moderate (0.40 ≤ r ≤ 0.69), strong (0.70 < r ≤ 0.89), and very strong (0.90 ≤ r) [30]. Statistical analyses were performed using JMP Pro 16 (SAS Institute Inc., Cary, NC, USA).

## 3. Results

### 3.1. Demographic Data and Clinical Characteristics

Post-stroke patients were assigned to different groups, with 13 in the high-risk group and 18 in the group with good balance ability (Table 1). There were no significant differences in basic demographic data, but clinical characteristics were significantly different between the high-risk group and the low-risk group (Table 1). The motor function of the lower limb was significantly lower in the high-risk group than in the low-risk group (FMA-L: *p* = 0.006, SIAS hip flexion test: *p* < 0.001, knee extension test: *p* = 0.006, foot tap test: *p* = 0.04, Table 1).

### 3.2. Trunk Angular Velocity and Acceleration in Walk–Turn–Walk Phase

The results of trunk angular velocity and acceleration during turning are shown in Table 2. The duration in the walk–turn–walk phase (χ^2^ = 31.21, *p* < 0.001, $\eta^{2}=0.77$) was significantly longer in the high-risk group than that in other groups (Table 2). The maximum value of ω_yaw_ (χ^2^ = 28.51, *p* < 0.001, $\eta^{2}=0.70$) in the high-risk group was significantly lower than that in other groups (Table 2). The minimum value of ω_pitch_ (χ^2^ = 9.80, *p* = 0.007, $\eta^{2}=0.25$) in the high-risk group was significantly lower than that in other groups (Table 2). The RMS values of ω_yaw_ (χ^2^ = 32.78, *p* < 0.001, $\eta^{2}=0.81$), α_V_ (χ^2^ = 21.17, *p* < 0.001, $\eta^{2}=0.50$), α_ML_ (χ^2^ = 21.19, *p* < 0.001, $\eta^{2}=0.50$) and α_AP_ (χ^2^ = 28.28, *p* < 0.001, $\eta^{2}=0.69$) in the high-risk group were significantly lower than that in other groups (Table 2). The comparisons of trunk angular velocity and acceleration in the SIT and STS phases are shown in Appendix A Table A2 and Table A3.

### 3.3. The Relationship Between the SIAS Score and Trunk Angular Velocity and Acceleration

The relationships between the SIAS score for lower extremity motor function and trunk movement during turning in post-stroke patients are shown in Table 3. The maximum ω_yaw_ (r = 0.591, *p* < 0.001) and the maximum α_AP_ (r = 0.416, *p* = 0.020) values in post-stroke patients were moderately correlated with hip flexion. The minimum ω _pitch_ (r = 0.520, *p* = 0.003) value in post-stroke patients was moderately correlated with hip flexion. The RMS of ω_yaw_ (r = 0.730, *p* < 0.001) in post-stroke patients was strongly correlated with hip flexion.

## 4. Discussion

This study investigated the differences in trunk movement during turning in post-stroke patients with high and low risks of falling and controls. We found decreased trunk rotation speed and trunk acceleration in the mediolateral direction in the high-risk group, which partially supports our hypothesis. On the other hand, increased trunk flexion velocity during turning in post-stroke patients with a high risk of falling did not support our hypothesis regarding angular velocity. To the best of our knowledge, this is the first study to reveal the characteristics of turning movement during turning in post-stroke patients with a high risk of falling.

The high-risk group showed a decrease in the trunk rotation speed and trunk acceleration in the antero-posterior direction and mediolateral direction during turning (Table 2). Previous studies have reported that post-stroke patients have poor balance ability compared to healthy subjects, resulting in longer turning movement times in the TUG test [14,31,32]. Our results indicate a similar trend with previous studies. Furthermore, the high-risk group exhibited the lowest trunk rotation speed and acceleration in the antero-posterior direction and mediolateral direction compared to the other groups (Table 2). These results indicate that in the high-risk group, both trunk movement speed during turning and weight shift in the unaffected side were reduced. In Figure 2, a clear trend of increased α_ML_ at the maximum ω_yaw_ during turning was observed in the low-risk group and control group. The peak Yaw value indicates the occurrence of the turning movement [33]. In contrast, a distinct increase in α_ML_ during turning was not found in the high-risk group. An increase in the trunk rotation speed is associated with an increase in centrifugal force, which causes the body to fall outward [34]. This suggests that a force inducing trunk lateral flexion to the outward direction is applied during turning. On the other hand, there were no significant differences in the maximum and minimum ω_roll_ values between the high-risk group and low-risk group. In other words, trunk lateral flexion during turning showed a similar trend between the two groups. This suggests that trunk rotation speed might be reduced to maintain stability in the mediolateral direction. Given that previous studies have demonstrated an association between trunk rotation speed and balance ability in post-stroke patients [30,31], our results suggest that post-stroke patients with poor balance ability may adopt a compensatory strategy of reducing trunk rotation and translational movement speed to enhance lateral trunk stability during turning. Thus, decreased movement speed and mediolateral instability during turning are associated with poor balance ability. These results suggest that a decrease in trunk rotation speed in the high-risk group will compensate for mediolateral stability during turning, thereby supporting our hypothesis.

Regarding ω_pitch_, which represents sagittal plane motion, the RMS value, indicating variability, was lower in the high-risk group than in the control group, suggesting a reduction in trunk flexion–extension angular velocity fluctuations during the walk–turn–walk phase (Table 2). The maximum ω_pitch_ was also lower in the high-risk group compared to the control group, indicating a lower trunk extension angular velocity in the high-risk group (Table 2). However, the minimum ω_pitch_ was lower in the high-risk group than in the other groups, indicating higher trunk flexion angular velocity in the high-risk group (Table 2). These results suggest that the trunk exhibited suppressed variability within a more flexion-dominant range of motion. Therefore, despite employing a strategy to reduce trunk flexion–extension variability during the walk–turn–walk phase, trunk flexion disturbance was observed in the high-risk group. A previous study reported that an increase in whole-body angular momentum in the sagittal plane was associated with a decrease in straight-line walking speed in post-stroke patients [35]. Our results show a decrease in trunk rotation speed and an increase in trunk disturbance in the sagittal plane during turning in the high-risk group (Table 2), which are similar to trends observed in a previous study. Furthermore, the generation of whole-body angular momentum in the sagittal plane during gait in stroke patients has been shown to be associated with walking speed. Consequently, not only during turning but also throughout the entire walk–turn–walk phase, trunk flexion instability occurred, and a characteristic trunk movement (i.e., ω_pitch_) synchronized with the turning action was observed. Trunk disturbances in the sagittal plane during walking are primarily compensated for by the hip joint, which maintains trunk stability [36,37]. The high-risk group exhibited more severe motor palsy, particularly in the hip joint, compared to the low-risk group (Table 1). Moreover, the relationship between hip flexion and trunk disturbance in the direction of trunk flexion suggests an association between lower extremity motor function and trunk disturbance (Table 3). These results suggest that the high-risk group lacks sufficient dynamic postural control through the hip joint during turning, thereby increasing trunk disturbance in the sagittal plane. Importantly, the high-risk group exhibited trunk disturbance in the sagittal plane during turning, even with a decreased movement speed intended to compensate for poor balance ability. This characteristic, contrary to our hypothesis, represents a new finding in our study. These findings suggest that the characteristics of turning movements in post-stroke patients depend on their balance ability, particularly the susceptibility of trunk flexion direction disturbance to balance ability.

These findings suggest that balance ability in post-stroke patients influence trunk movement during turning. While a previous study has reported improved balance ability with the recovery of motor function [38], our study found an association between hip motor paralysis and the characteristics of trunk movement during turning. This indicated that motor function of the paretic lower limb is also related to trunk movement during turning, suggesting a link between lower limb motor function and trunk control during turning. The results of this study indicate that rehabilitation focusing on improving physical function (i.e., paretic lower limb motor function), in addition to controlling trunk lateral and flexion sway during turning, may contribute to the safe execution of turning movements frequently encountered in daily life.

This study has several limitations. First, participants with cognitive impairment were excluded. Given the known relationship between cognitive impairment and balance ability, including such participants could potentially yield different results [39]. Second, the use of canes and orthoses was not restricted. Canes can assist in stabilizing posture during movement [40,41]. Third, this study divided the TUG test into three subcomponents: the STS, walk–turn–walk, and SIT phases. Further division of the walking and turning phases could allow for a more detailed analysis of trunk movement during turning. The action of changing direction from linear walking imposes centrifugal forces on the body [34]. Previous research comparing the center of mass (COM) trajectory during the TUG test between stroke patients and healthy individuals has reported a significant lateral deviation of the COM trajectory in stroke patients, specifically during the turning phase [42]. Thus, by clearly separating the turning movement phase from straight-line walking, it may become possible to elucidate the characteristics of lateral movements specifically associated with turning. Fourth, the measurement reliability of the iPhone 12 mini used in this study has not been directly verified in the existing literature to the best of our knowledge. Therefore, we calculated the intraclass correlation coefficient (ICC). The results indicate compromised intra-rater reliability for minimum ω_yaw_ (Appendix A Table A1). However, the yaw values are not included in the main results. Future research utilizing validated inertial measurement units (IMUs) may reveal more detailed characteristics of trunk movement. Fifth, this study was conducted using a comfortable speed in the TUG. While maximum movement speed is associated with balance ability, other studies have reported that individuals with good balance ability exhibit less associated movement speed [43]. Therefore, this study employed a comfortable speed in the TUG test. Sixth, this study had a small sample size. Therefore, effect size and statistical power were assessed, and the main results show adequate statistical power. However, these results should be interpreted with caution as they do not include post-stroke patients with cognitive impairment and are limited to those who can undergo the TUG assessment, which may restrict their generalizability to all post-stroke patients. Simultaneous measurements of trunk kinematics, lower limb kinematics, and muscle activity during turning movements in future research may yield a more comprehensive understanding of trunk control in post-stroke patients during this challenging task.

## 5. Conclusions

This study clarified the characteristics of trunk movement during turning in post-stroke patients based on differences in balance ability. Trunk rotation and translational speed and mediolateral acceleration were the lowest in post-stroke patients with a high risk of falling compared to the other groups and were correlated with balance ability in post-stroke patients. On the other hand, the post-stroke patients with a high risk of falling exhibited greater trunk disturbance in the sagittal plane during turning compared to other groups. Furthermore, trunk disturbance in the sagittal plane during turning was correlated with hip joint function. These findings indicate that trunk disturbance in the sagittal plane during turning is characteristic of poor balance ability in post-stroke patients. This may suggest the need for a trunk control strategy focusing on the hip joint of the affected side during turning to enhance trunk stability alongside physical function.

## Figures and Tables

**Table 1:** Characteristics of post-stroke patients and controls.

	High-Risk Group	Low-Risk Group	Control	*p*-Value
Number	13	18	10	
Age (years) ^ac^	69.38 (12.44)	71.22 (8.50)	65.90 (11.57)	0.67
Sex (male/female) ^be^	7/6	12/6	6/4	0.76
Height (m) ^ac^	1.57 (0.08)	1.62 (0.01)	1.63 (8.00)	0.23
Weight (kg) ^ac^	55.01 (11.46)	51.56 (7.50)	60.27 (9.49)	0.11
Time post-stroke (days) ^ad^	64.84 (36.20)	78.61 (40.21)	-	0.32
Diagnosis (hemorrhage/infarction) ^be^	8/5	5/13	-	0.11
Paretic side (right/left) ^be^	3/10	10/8	-	0.06
Cane (T-cane/none) ^be^	11/2	4/14	0/10	<0.001
Orthosis (ankle foot orthosis/none) ^be^	8/5	1/17	0/10	<0.001
BBS ^ad^	35.30 (5.52)	50.94 (3.47)	-	<0.001
FMA-L ^ad^	24.15 (3.05)	27.94 (3.05)	-	0.006
SIAS (1/2/3/4/5) ^be^				
Hip flexion test	0/1/2/10/0	0/1/0/2/15	-	<0.001
Knee extension test	0/3/4/4/2	0/0/0/6/12	-	0.006
Ankle dorsiflexion test	2/0/4/3/4	0/0/1/6/11	-	0.04
TIS ^ad^	13.62 (4.10)	16.78 (3.02)	-	0.07

^a^ mean (standard deviation), ^b^ number, ^c^ Kruskal–Wallis test, ^d^ Willcoxon rank sum test, ^e^ Chi-square test. BBS: Berg Balance Scale, FMA-L: Fugl-Meyer Assessment of Lower extremity, SIAS: Stroke Impairment Assessment Set, TIS: Trunk Impairment Scale.

**Table 2:** Comparison of inertial measurement unit data during turn movements between post-stroke patients and controls.

	High-Risk Group	Low-Risk Group	Control	Effect Size ($\eta^{2}$)	Power (1 − β)
Time (s)	14.17 (4.71) ^ac^	6.54 (2.29) ^ab^	3.31 (0.97) ^bc^	0.77	0.99
Maximum					
ω_yaw_ (deg/s)	65.60 (15.56) ^ab^	103.38 (26.93) ^ac^	174.29 (26.47) ^bc^	0.70	1.00
ω_pitch_ (deg/s)	15.65 (4.07) ^b^	15.79 (3.58)	25.28 (12.92) ^b^	0.13	0.40
ω_roll_ (deg/s)	10.22 (3.70)	9.16 (4.04)	16.70 (13.95)		
α_V_ (m/s^2^)	3.60 (0.85) ^b^	3.70 (1.00) ^c^	6.67 (3.29) ^bc^	0.32	0.57
α_ML_ (m/s^2^)	4.19 (1.25) ^b^	5.37 (2.09) ^c^	9.20 (2.29) ^bc^	0.43	1.00
α_AP_ (m/s^2^)	5.12 (2.10) ^ab^	7.12 (1.95) ^ac^	11.91 (5.42) ^bc^	0.40	0.74
Minimum					
ω_yaw_ (deg/s)	−16.39 (6.37)	−14.27 (8.40)	−10.72 (12.95)		
ω_pitch_ (deg/s)	−17.65 (6.78) ^ab^	−11.04 (4.95) ^a^	−11.12 (10.26) ^b^	0.25	0.36
ω_roll_ (deg/s)	−12.62 (6.54) ^b^	−16.14 (12.61) ^c^	−38.13 (28.63) ^bc^	0.19	0.49
α_V_ (m/s^2^)	−5.79 (2.38) ^b^	−6.24 (2.17) ^c^	−10.45 (4.41) ^bc^	0.22	0.66
α_ML_ (m/s^2^)	−3.45 (1.09)	−3.73 (0.84)	−5.27 (2.19)		
α_AP_ (m/s^2^)	−2.47 (0.62) ^ab^	−3.25 (0.69) ^ac^	−4.83 (0.98) ^bc^	0.57	1.00
RMS					
ω_yaw_ (deg/s)	25.50 (5.02) ^ab^	48.00 (12.15) ^ac^	83.65 (14.16) ^bc^	0.81	1.00
ω_pitch_ (deg/s)	7.02 (2.20) ^b^	6.51 (1.57) ^c^	13.26 (7.82) ^bc^	0.29	0.50
ω_roll_ (deg/s)	5.37 (2.60) ^b^	8.13 (5.78) ^c^	20.04 (11.89) ^bc^	0.37	0.76
α_V_ (m/s^2^)	1.14 (0.32) ^ab^	1.87 (0.80) ^ac^	3.21 (1.38) ^bc^	0.50	1.00
α_ML_ (m/s^2^)	1.07 (0.39) ^ab^	1.42 (0.46) ^ac^	2.71 (0.82) ^bc^	0.50	0.99
α_AP_ (m/s^2^)	1.07 (0.27) ^ab^	1.68 (0.43) ^ac^	2.92 (0.89) ^bc^	0.69	0.99

^a^ Significant difference between high-risk group and low-risk group, *p* < 0.05; ^b^ significant difference between high-risk group and control, *p* < 0.05; ^c^ significant difference between low-risk group and control, *p* < 0.05. RMS: Root mean square.

**Table 3:** The relationship between motor function and inertial measurement unit data during turn movement.

	Hip Flexion Test		Knee Extension Test		Foot Pat Test	
	Correlation Coefficient (r)	*p*-Value	Correlation Coefficient (r)	*p*-Value	Correlation Coefficient (r)	*p*-Value
Maximum ω_yaw_	0.591	<0.001 *	0.481	0.006	0.054	0.774
Maximum α_AP_	0.416	0.020 *	0.220	0.234	−0.095	0.609
Minimum ω_pitch_	0.520	0.003 *	0.383	0.033 *	0.188	0.312
Minimum α_AP_	−0.511	0.003 *	−0.426	0.017 *	−0.006	0.975
RMS ω_yaw_	0.730	<0.001 *	0.600	<0.001 *	0.244	0.185
RMS α_V_	0.550	0.001 *	0.337	0.064	0.007	0.971
RMS α_ML_	0.522	0.003 *	0.438	0.014 *	−0.080	0.670
RMS α_AP_	0.571	0.001 *	0.464	0.009 *	0.024	0.899

* *p* < 0.05, RMS: root mean square. Angular velocity around vertical axis (ω_yaw_), mediolateral axis (ω_pitch_), antero-posterior axis (ω_roll_). Acceleration along antero-posterior (α_AP_), vertical (α_V_), and mediolateral (α_ML_) axes. Correlation coefficients: weak, 0.10 ≤ r ≤ 0.39; moderate, 0.40 ≤ r ≤ 0.69; strong, 0.70 ≤ r ≤ 0.89; very strong, 0.90 ≤ r.

## Data Availability

The data, including graphs, within this paper are available from the corresponding author upon reasonable request.

## Appendix A

**Table A1:** Intraclass correlation coefficients for the walk–turn–walk phase.

Walk–Turn–Walk	ICC (1, 3)		
	Maximum	Minimum	RMS
ω_yaw_	0.85	0.16	0.97
ω_pitch_	0.82	0.53	0.94
ω_roll_	0.73	0.94	0.97
α_Z_	0.78	0.74	0.62
α_ML_	0.85	0.68	0.92
α_AP_	0.85	0.60	0.92

Intraclass correlation coefficients values < 0.5 indicate poor reliability, values between 0.5 and 0.75 indicate moderate reliability, values between 0.75 and 0.90 indicate good reliability, and values greater than 0.90 indicate excellent reliability.

**Table A2:** Comparison of sit-to-stand movement using IMU data between post-stroke patients and controls.

	High-Risk Group	Low-Risk Group	Control	Effect Size ($\eta^{2}$)	Power (1 − β)
Time (s)	2.43 (0.52) ^ac^	1.99 (0.24) ^a^	1.90 (0.36) ^c^	0.18	0.65
Maximum					
ω_yaw_	10.38 (6.70) ^b^	11.56 (6.52) ^c^	33.73 (22.63) ^bc^	0.20	0.66
ω_pitch_	31.44 (11.97) ^b^	38.17 (9.79) ^c^	51.82 (10.98) ^bc^	0.31	0.95
ω_roll_	11.63 (4.53)	10.82 (5.29)	16.00 (5.14)		
α_V_	1.93 (0.57) ^ab^	3.27 (1.26) ^ac^	7.09 (3.62) ^bc^	0.60-	0.86
α_ML_	1.39 (0.64) ^ab^	2.31 (1.12) ^ac^	4.97 (2.31) ^bc^	0.44	0.91
α_AP_	1.68 (0.81) ^ab^	4.03 (2.23) ^ac^	10.78 (7.19) ^bc^	0.62	0.76
Minimum					
ω_yaw_	−15.12 (4.66) ^b^	−15.60 (6.99) ^c^	−27.28 (13.56) ^bc^	0.19	0.53
ω_pitch_	−66.00 (15.59) ^a^	−88.39 (12.18) ^ac^	−100.72 (21.05) ^c^	0.40	0.95
ω_roll_	−7.50 (1.75) ^b^	−7.86 (3.87)	−13.91 (7.57) ^b^	0.18	0.48
α_V_	−2.37 (1.41) ^ab^	−3.66 (1.40) ^ac^	−7.75 (4.51) ^bc^	0.45	0.71
α_ML_	−1.40 (0.59) ^b^	−1.71 (0.90) ^c^	−4.32 (2.16) ^bc^	0.40	0.86
α_AP_	−1.43 (0.50) ^ab^	−2.58 (0.93) ^ac^	−6.69 (5.95) ^bc^	0.65	0.45
RMS					
ω_yaw_	8.15 (2.29) ^b^	7.95 (2.43)	14.48 (6.32) ^bc^	0.23	0.67
ω_pitch_	34.58 (8.00) ^ab^	45.43 (5.17) ^a^	52.16 (10.22) ^b^	0.45	0.96
ω_roll_	6.20 (2.17)	6.26 (2.92)	9.16 (3.17)		
α_V_	0.78 (0.27) ^ab^	1.41 (0.49) ^ac^	2.76 (1.08) ^bc^	0.68	0.97
α_ML_	0.48 (0.20) ^b^	0.69 (0.32) ^c^	1.45 (0.62) ^bc^	0.46	0.93
α_AP_	0.58 (0.18) ^ab^	1.20 (0.51) ^ac^	2.63 (1.12) ^bc^	0.68	0.97

^a^: Significant difference between high-risk group and low-risk group, *p* < 0.05; ^b^: significant difference between high-risk group and control, *p* < 0.05; ^c^: significant difference between low-risk group and control, *p* < 0.05. RMS: Root mean square.

**Table A3:** Comparison of stand-to-sit movement using IMU data between post-stroke patients and controls.

	High-Risk Group	Low-Risk Group	Control	Effect Size ($\eta^{2}$)	Power (1 − β)
Time (s)	4.56 (0.91) ^ac^	3.38 (0.61) ^ab^	2.13 (0.55) ^bc^	0.66	0.99
Maximum					
ω_yaw_	73.49 (17.39) ^ab^	110.08 (28.08) ^ac^	193.60 (25.78) ^bc^	0.71	1.00
ω_pitch_	59.28 (10.95) ^ab^	73.08 (14.86) ^ac^	87.84 (15.63) ^bc^	0.36	0.97
ω_roll_	11.82 (7.55)	10.29 (6.61)	10.35 (15.10)		
α_V_	3.76 (1.43)	3.79 (1.29) ^c^	5.17 (1.81) ^c^	0.12	0.43
α_ML_	3.53 (1.26) ^b^	4.15 (1.37)	5.88 (2.00) ^b^	0.18	0.69
α_AP_	4.73 (2.49)	6.20 (1.79)	7.66 (3.41)		
Minimum					
ω_yaw_	−4.26 (6.51)	−9.02 (12.42)	−8.13 (7.21)		
ω_pitch_	−28.02 (9.77) ^b^	−36.60 (9.91)	−49.06 (11.86) ^b^	0.35	0.96
ω_roll_	−23.12 (10.63)	−30.46 (14.02)	−48.09 (33.85)		
α_V_	−7.07 (4.06)	−6.10 (2.68)	−6.91 (2.54)		
α_ML_	−3.08 (1.21)	−3.12 (0.78)	−3.97 (1.29)		
α_AP_	−4.24 (2.21)	−3.56 (1.20)	−3.33 (0.82)		
RMS					
ω_yaw_	37.97 (11.34) ^ab^	54.14 (12.41) ^ac^	98.65 (16.03) ^bc^	0.63	1.00
ω_pitch_	22.78 (4.33) ^ab^	31.52 (6.60) ^ac^	44.95 (9.56) ^bc^	0.60	1.00
ω_roll_	11.33 (5.41) ^b^	17.00 (7.77)	28.15 (17.29) ^b^	0.17	0.51
α_V_	1.27 (0.56) ^b^	1.42 (0.44) ^c^	2.24 (0.68) ^bc^	0.28	0.89
α_ML_	1.03 (0.38) ^b^	1.20 (0.28) ^c^	1.69 (0.47) ^bc^	0.29	0.84
α_AP_	1.00 (0.33) ^ab^	1.35 (0.38) ^ac^	1.96 (0.62) ^bc^	0.39	0.90

^a^: Significant difference between high-risk group and group with good balance ability, *p* < 0.05; ^b^: significant difference between high-risk group and control, *p* < 0.05; ^c^: significant difference between low-risk group and control, *p* < 0.05. RMS: Root mean square.
